# Enhanced frequency and potential mechanism of B regulatory cells in patients with lung cancer

**DOI:** 10.1186/s12967-014-0304-0

**Published:** 2014-11-11

**Authors:** Jiebai Zhou, Zhihui Min, Ding Zhang, William Wang, Francesco Marincola, Xiangdong Wang

**Affiliations:** Department of Pulmonary Medicine, Shanghai, China; Biomedical Research Center, Zhongshan Hospital, Shanghai, China; Fudan University Center for Clinical Bioinformatics, Shanghai, China; Department of Biomedical Sciences, UCL, London, UK; Sidra Medical and Research Centre, Doha, Qatar

**Keywords:** Regulatory T cells, Regulatory B cells, Lung cancer, Lymphocytes, Microenvironment

## Abstract

**Background:**

Regulatory T cells (Tregs) and B cells (Bregs) play an important role in the development of lung cancer. The present study aimed to investigate the phenotype of circulating Tregs and Bregs in patients with lung cancer and explore potential mechanism by which lung cancer cells act on the development of both.

**Methods:**

Patients with lung cancer (n = 268) and healthy donors (n = 65) were enrolled in the study. Frequencies of Tregs and Bregs were measured by flow cytometry with antibodies against CD4, CD25, CD127, CD45RA, CD19, CD24, CD27 and IL-10 before and after co-cultures. qRT-PCR was performed to evaluate the mRNA levels of RANTES, MIP-1α, TGF-β, IFN-γ and IL-4.

**Results:**

We found a lower frequency of Tregs and a higher frequency of Bregs in patients with lung cancer compared to healthy donors. Co-culture of lung cancer cells with peripheral blood mononuclear cells could polarize the lymphocyte phenotype in the similar pattern. Lipopolysaccharide (LPS)-stimulated lung cancer cells significantly modulated regulatory cell number and function in an *in vitro* model.

**Conclusion:**

We provide initial evidence that frequencies of peripheral Tregs decreased or Bregs increased in patients with lung cancer, which may be modulated directly by lung cancer cells. It seems cancer cells *per se* plays a crucial role in the development of tumor immunity.

**Electronic supplementary material:**

The online version of this article (doi:10.1186/s12967-014-0304-0) contains supplementary material, which is available to authorized users.

## Introduction

Lung cancer is the most prevalent malignant tumor and the leading cause of cancer-associated morbidity and mortality [1]. Over 1.4 million people were diagnosed with lung cancer in 2004 and about 1.3 million people die of lung cancer each year, according to the Global Burden of Disease study [2]. Both tumor characteristics immune responses of patients with lung cancer could affect tumor development [3]. Growing evidence has proposed an opposing role of the immune system in fostering tumor growth, in spite of the considerable evidence indicating that the immune system can recognize and destroy tumor cells [4-6].

Regulatory T cells (Tregs) are a subpopulation of T cells with immune suppressive function. Recent studies demonstrated elevated percentages of Tregs in the total T cell population isolated from tumor tissues or peripheral blood in a variety of cancers, including lung cancer [7-9]. The accumulation of Tregs might be associated with advanced tumor growth and poor prognosis in lung cancer [10-12]. Regulatory B cells (Bregs) were also found to play a regulatory role in immune responses via the production of regulatory cytokines, such as IL-10 and TGF-β, and express inhibitory molecules to suppress pathogenic T cells and autoreactive B cells in a cell-to-cell contact-dependent manner [13,14]. The absence or loss of Bregs may exacerbate disease symptoms in autoimmune diseases [15], chronic inflammatory diseases [16], or promot tumor progression. It was reported that Bregs played a critical role in pulmonary metastasis of breast cancer through inducing recruitment and expansion of Tregs [17]. In developing tumors anti-tumorigenic and pro-tumorigenic immune and inflammatory mechanisms coexist, and the net effect of them affects tumor development [18].

However, there are few studies on the role of Bregs in lung cancer and the potential interaction of lung cancer cells on the development of Treg and Breg. The present study aimed to investigate the phenotype of circulating Tregs and Bregs in patients with lung cancer and explore potential mechanism by which lung cancer cells act on the antitumor immunity.

## Patients and methods

### Blood samples collection

Peripheral blood samples were collected upon patient admission before any therapeutic intervention. The diagnosis of lung cancer was made on the basis of imaging or biopsy examination (n = 268). Control samples were obtained from healthy donors (n = 65). All blood samples were collected after informed consent was given. The present study was approved by the Ethical Evaluation Committee of Zhongshan Hospital.

### Cell isolation and culture

Peripheral blood mononuclear cells (PBMC) were isolated as previously described [19]. In brief, whole blood samples were overlaid onto Ficoll separation media (Tianjin Haoyang Biological Manufacture, China) after 1:1 dilution with Hank’s Balanced Salted Solution (Gibco, CA, USA). PBMCs were centrifuged for 15 min at × 2800 rpm, collected at the plasma interface and washed thrice after centrifugation at × 1500 rpm for 10 min. Human alveolar adenocarcinoma cell line A549, which were from our research center, and the isolated PBMCs were cultured in DMEM (high glucose, Hyclone, USA), supplemented with 10% FBS (Hyclone, USA), 100U/ml penicillin, and 100 μg/ml streptomycin at 37°C in a 5% CO_2_, 95% air environment in humidified incubators.

### Transwell experiment

Twelve-well transwell chambers with a 0.4 μm porous membrane (Corning-Costar, USA) were used. A549 cells (5 × 10^5^/well) were plated underneath the transwell chamber and stimulated with LPS, and then 0.5 ml of PBMC (2 × 10^6^/ml) was added to the inner chamber at 24 hrs after LPS stimulation. After co-culturing for 48 hrs, PBMCs were harvested and stained by flow cytometry, while A549 cells were harvested and prepared for quantitative real time polymerase chain reaction (qRT-PCR). To investigate the role of LPS-related signal pathway, A549 cells were pretreated with NF-κB inhibitor PDTC at 10, 50, 100, 300, or 500 μM for 4 hrs.

### Flow cytometry analysis

Flow cytometry analysis was conducted by FACS Aria II flow cytometry (BD Bioscience, USA). For surface staining, suspensions of PBMCs were stained on ice using predetermined optimal concentrations of each antibody for 30 min, and fixed using fixation buffer (BD PharMingen, USA). Tregs identified with CD4^+^CD25^+^CD127^−^ expression were stained with human regulatory T cell Cocktail (BD PharMingen, USA) [20] and Bregs identified with CD19^+^CD24^hi^CD27^+^ expression were stained with human anti-CD19, human anti-CD24, and human anti-CD27 (BD PharMingen, USA) [21]. Intracellular IL-10 analysis was performed by flow cytometry, as described previously [22]. Briefly, cells were resuspended (2 × 10^6^ cells/ml) in medium and stimulated with ODN2006 (10 μg/ml; Sangon Biotech, Shanghai, China) for 24 hrs with leukocyte activation cocktail (2 μl/ml; BD GolgiPlug™, BD Pharmingen, USA) added during the final 5 hrs before staining. After surface staining, cells were fixed, permeabilized using a Cytofix/Cytoperm™ Kit (BD PharMingen, USA), and stained with human anti-IL10 (BD PharMingen, USA) according to the manufacturer’s instructions. Results are expressed as frequency of Tregs or Bregs.

### Quantitative real time polymerase chain reaction (qRT-PCR)

RNA extraction was performed using the TRIZOL™LS reagent (Invitrogen, Carlsbad, CA). cDNA was prepared using PrimeScript® RT reagent Kit (Takara, Shiga, Japan) following standard protocols. qRT-PCR was performed using SYBR® Premix Ex Taq™ (Takara, Shiga, Japan) on the ABI PRISM 7900 real-time PCR system (Applied Biosystems, Foster City, CA). All samples were run in triplicate. Results are shown as relative target mRNA levels.

#### Experimental design

1. To evaluate the frequency of peripheral Tregs and Bregs in patients with lung cancer, 268 patients were recruited from 800 patients with lung cancer under the restricted criteria.
2. To investigate the role of inflammation in shaping the phenotype of PBMC. To reveal the role that cell-cell-contact or cytokines play in phenotype alterations, A549 cells were stimulated with LPS at 10, 100, 1000 ng/ml or vehicle for 24 hrs, and LPS-stimulated A549 cells as activated LC cells and their supernatant as activated medium were then harvested. PBMCs from healthy donors were co-cultured with the harvested activated or non-activated A549 cells and medium for 48 hrs, respectively. The control group was PBMC from healthy donors without co-culture. Treg and Breg frequencies were enumerated by flow cytometry (Additional file 1: Figure S1A).
3. To reveal indirect effects of activated lung cancer cells on PBMC phenotypes and to investigate whether continuous stimulation by LPS will bears different effects on PBMC phenotype, A549 cells were planted in the lower chamber of the transwell and stimulated with LPS at 100 and 500 ng/ml or vehicle for 24 hrs. PBMCs from healthy donors were then added to the upper chamber of the transwell for co-culture for 48 hrs. The control group was PBMC from healthy donors without co-culture. Treg and Breg frequencies were enumerated by flow cytometry. The co-cultured A549 cells were also harvested for qPCR for mRNA expression of RANTES and MIP-1α, while the co-cultured PBMCs were harvested for mRNA expression of TGF-β, IFN-γ, and IL-4. The control group was A549 cell or PBMC from healthy donors without co-culture (Additional file 1: Figure S1B).
4. To investigate the role of LPS-related NF-κB signal pathway in the activation of lung cancer cells. A549 cells were planted in the lower chamber of the transwell and pretreated with NF-κB inhibitor PDTC at 10, 50, 100, 300, 500 μM or vehicle for 4 hrs, and then washed with fresh medium. After then, PDTC pre-treated A549 cells were stimulated with LPS at 500 ng/ml for 24 hrs and PBMCs from healthy donors were added to the upper chamber of the transwell for co-culture for 48 hrs. Treg frequencies were enumerated by flow cytometry (Additional file 1: Figure S1C).
5. To investigate the role of inflammation-activated lung cancer cells in phenotype alterations of PBMC obtained from patients with lung cancer and the phenotype difference between lung cancer patients and healthy individuals. A549 cells were stimulated with LPS at 100 and 500 ng/ml for 24 hrs, and LPS-stimulated A549 cells and their supernatant were then harvested. PBMC from lung cancer patients were co-cultured with harvested LPS-stimulated A549 cells and their supernatant for 48 hrs, respectively. The control group was PBMC from lung cancer patients without co-culture. Treg and Breg frequencies were enumerated by flow cytometry (Additional file 1: Figure S1D).

### Statistical analysis

All values were expressed as mean ± SEM. Statistical analysis was performed using SPSS software (SPSS 20.0; SPSS Inc; Chicago, IL). Frequencies of peripheral Tregs and Bregs among groups were analyzed with one-way ANOVA, followed by an unpaired student’s *t*-test. *P* <0.05 was considered as statistically significant.

## Results

**Figure 1:**
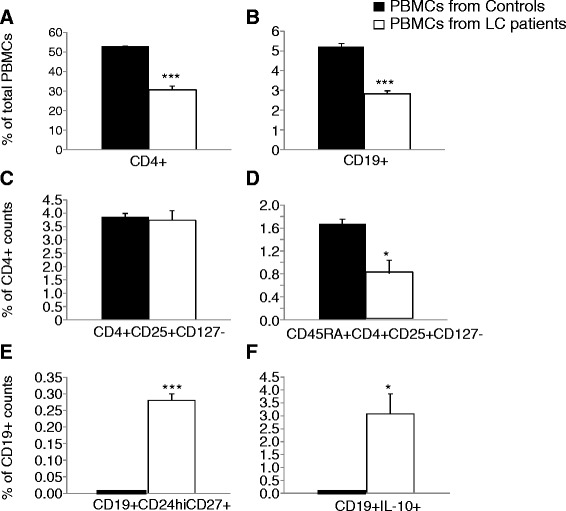
**Alteration of peripheral frequencies of regulatory lymphocytes in patients with lung cancer. A**: peripheral frequency of CD4^+^ T cells in total peripheral blood mononuclear cells (PBMCs), **B**: peripheral frequency of CD19^+^ B cells in total PBMCs, **C**: peripheral frequency of Tregs in CD4^+^ T cells, **D**: peripheral frequency of CD45RA^+^Tregs in CD4^+^ T cells, **E**: peripheral frequency of CD19^+^CD24^hi^CD27^+^ B cells in CD19^+^ B cells, and **F**: peripheral frequency of CD19^+^IL-10^+^ B cells in CD19^+^ B cells. * and *** stand for p value less than 0.05 and 0.001, as compared to healthy control, respectively.

Frequencis of CD4^+^T cells and CD19^+^B cells in PBMCs from patients with lung cancer significantly decreased as compared with healthy individuals (P <0.001, Figure 1A and B, respectively). The frequency of peripheral Tregs (CD4^+^CD25^+^CD127^−^) in CD4^+^T cells and frequency of naïve Tregs (CD45RA^+^CD4^+^CD25^+^CD127^−^) in CD4^+^T cells from lung cancer patients was significantly lower than in the healthy (P <0.05; Figure 1C and D, respectively). The frequency of peripheral Bregs (CD19^+^CD24^hi^CD27^+^) and CD19^+^IL-10^+^B cells in CD19^+^B cells in lung cancer patients were significantly higher than in the healthy, as shown on Figure 1E and F (P <0.001 and 0.05, respectively).

**Figure 2:**
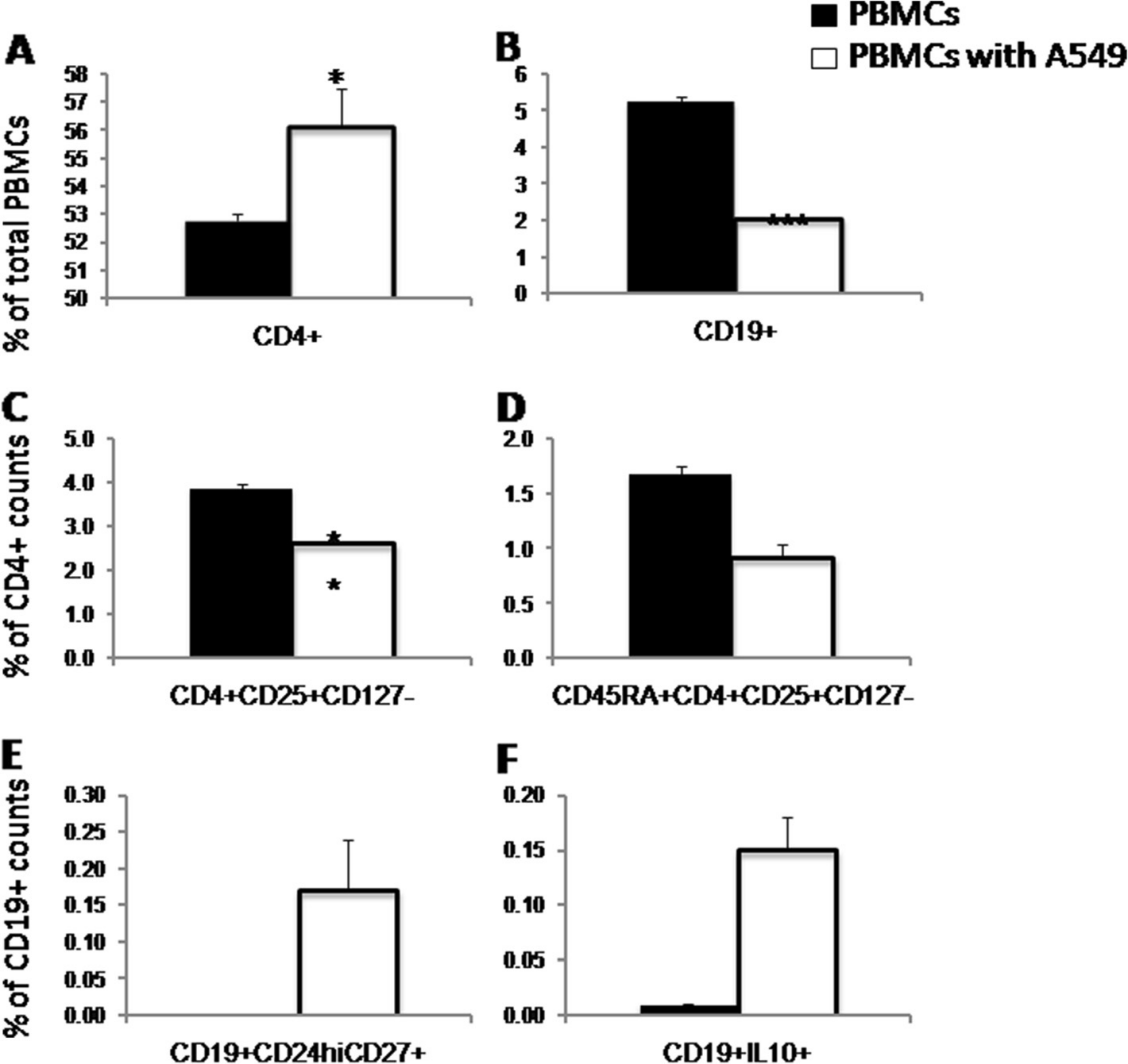
**Direct effects of lung cancer cells on peripheral blood mononuclear cells (PBMCs) measured during the co-culture of PBMCs from healthy donors with lung cancer cells (A549). A**: Frequency of CD4^+^ T cells in total PBMCs, **B**: Frequency of CD19^+^ B cells in total PBMCs, **C**: Frequency of Tregs in CD4^+^ T cells, **D**: Frequency of CD45RA^+^Tregs in CD4^+^ T cells, **E**: Frequency of CD19^+^CD24^hi^CD27^+^ B cells in CD19^+^ B cells, and **F**: Frequency of CD19^+^IL-10^+^ B cells in CD19^+^ B cells. *, **, and *** stand for p value less than 0.05, 0.01, and 0.001, as compared to PBMCs alone, respectively.

The frequency of CD4^+^T cells significantly increased (P <0.05; Figure 2A), while the frequency of CD19^+^B cells, Tregs and CD45RA^+^Tregs decreased after co-culture with A549 cells (Figure 2B,C and D, respectively). As shown in Figure 2E, the background frequency of CD19^+^CD24^hi^CD27^+^B cells was below the threshold for quantification by flow cytometry analysis. The frequency of B cells spontaneously expressing IL-10 was only 0.01% (Figure 2F). After co-culture with A549 cells, the proportion of CD19^+^CD24^hi^CD27^+^ and CD19^+^IL-10^+^ B cells elevated above background (Figure 2E and F, respectively).

**Figure 3:**
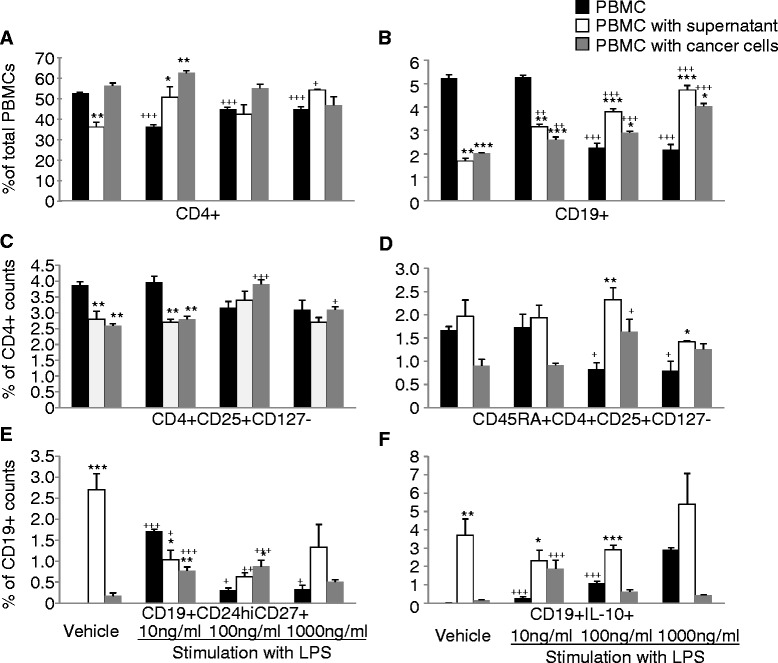
**Direct effects of activated lung cancer cells or their mediators on peripheral blood mononuclear cells (PBMCs) measured during the co-culture of PBMCs from healthy donors with lung cancer cells (A549) or the supernatant pre-stimulated with lipopolysaccharide (LPS), respectively.** A549 cells were stimulated with LPS at 10, 100, 1000 ng/ml or vehicle for 24 hrs, and then LPS-stimulated A549 cells as activated LC cells and their supernatant as activated medium were harvested. After then, PBMCs from healthy donors were co-cultured with the harvested activated or non-activated A549 cells and medium for 48 hrs, respectively. The control group was PBMC from healthy donors without co-culture. **A**: Frequency of CD4^+^ T cells in total PBMCs, **B**: Frequency of CD19^+^ B cells in total PBMCs, **C**: Frequency of Tregs in CD4^+^ T cells, **D**: Frequency of CD45RA^+^Tregs in CD4^+^ T cells, **E**: Frequency of CD19^+^CD24^hi^CD27^+^ B cells in CD19^+^ B cells, and **F**: Frequency of CD19^+^IL-10^+^ B cells in CD19^+^ B cells. +, ++, and +++ stand for p values less than 0.05, 0.01, and 0.001, as compared with corresponding controls including vehicle-stimulated PBMC, PBMC co-culture with vehicle-stimulated A549 supernatant, or PBMC co-culture with vehicle-stimulated A549 cells, respectively. *, **, and *** stand for p values less than 0.05, 0.01, and 0.001, as compared with corresponding LPS-stimulated PBMC, respectively.

The frequency of CD4^+^T cells significantly increased after co-culture either with LPS-stimulated A549 cells or the conditioned supernatant (Figure 3A). The frequency of CD19^+^B cells increased in a LPS-concentration-dependent manner (Figure 3B). The frequencies of Tregs or CD45RA^+^Tregs reached to the highest level when LPS concentration was 100 ng/ml (Figure 3C and D). The alterations of frequencies of CD45RA^+^Tregs were similar to those of Tregs. LPS-stimulation-conditioned supernatant had more effect on the CD45RA^+^Tregs phenotype than LPS-stimulated A549 cells *per se*. Frequencies of CD19^+^CD24^hi^CD27^+^B cells were significantly lower after co-culture with conditioned supernatant, as compared with the control group (Figure 3E). The frequency of CD19^+^IL-10^+^B cells reached to the highest level when co-culture with the conditioned supernatant when LPS concentration was 1000 ng/ml (Figure 3F). Co-culture with LPS-stimulated A549 cells significantly increases the proportion of CD19^+^CD24^hi^CD27^+^ and CD19^+^IL-10^+^ B cells under all concentrations of LPS (Figure 3E and F, respectively).

**Figure 4:**
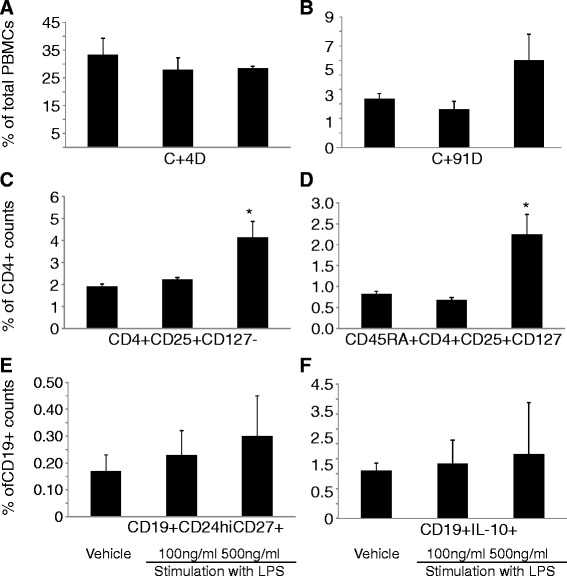
**Indirect effects of lung cancer cells on peripheral blood mononuclear cells (PBMCs) measured during the co-culture of lung cancer cells (A549) with PBMCs from healthy donors in a transwell model.** A549 cells were planted in the lower chamber of the transwell and stimulated with LPS at 100 and 500 ng/ml or vehicle for 24 hrs. PBMCs from healthy donors were then added to the upper chamber of the transwell for co-culture for 48 hrs. **A**: frequency of CD4^+^ T cells in total PBMCs, **B**: frequency of CD19^+^ B cells in total PBMCs, **C**: frequency of Tregs in CD4^+^ T cells, **D**: frequency of CD45RA^+^Tregs in CD4^+^ T cells, **E**: frequency of CD19^+^CD24hiCD27^+^ B cells in CD19^+^ B cells; **(F)** Frequency of CD19^+^IL-10^+^ B cells in CD19^+^ B cells. * stand for p values less than 0.05, as compared with the control group with PBMC from healthy donors without co-culture.

Study on the co-culture of A549 cells with PBMCs in the presence of continuous stimulation with LPS demonstrated LPS stimulation significantly decreased frequencies of CD4^+^T cells (Figure 4A), while increased frequencies of CD19^+^B cells at 500 ng/ml of LPS (Figure 4B). Figure 4C showed significantly increased frequencies of Tregs in a concentration-dependent pattern, while a decreased frequency of CD45RA^+^Tregs at 100 ng/ml of LPS. The frequency significantly increased to the highest level at 500 ng/ml of LPS (P <0.05; Figure 4D). A concentration-dependent increase in frequencies of CD19^+^CD24^hi^CD27^+^ and CD19^+^IL-10^+^ B cells was noted in CD19^+^B cells (Figure 4E and F, respectively).

**Figure 5:**
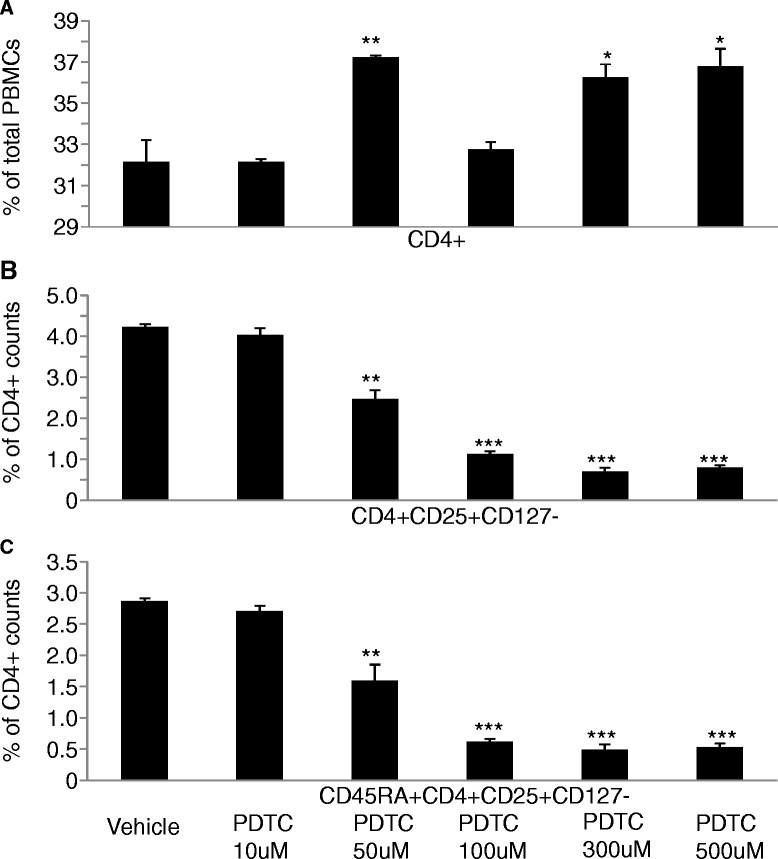
**The role of LPS-related NF-κB signal pathway in the activation of lung cancer cells (A549).** A549 cells were planted in the lower chamber of the transwell and pretreated with NF-κB inhibitor PDTC at 10, 50, 100, 300, 500 μM or vehicle for 4 hrs, and then washed with fresh medium. PDTC pre-treated A549 cells were stimulated with LPS at 500 ng/ml for 24 hrs and PBMCs from healthy donors were then added to the upper chamber of the transwell for co-culture for 48 hrs. **A**: frequency of CD4^+^ T cells in total PBMCs, **B**: frequency of Tregs in CD4^+^ T cells, and **C**: frequency of CD45RA^+^Tregs in CD4^+^ T cells. *, **, and *** stand for p values less than 0.05, 0.01, and 0.001, as compared with controls pretreated with vehicle, respectively.

**Figure 6:**
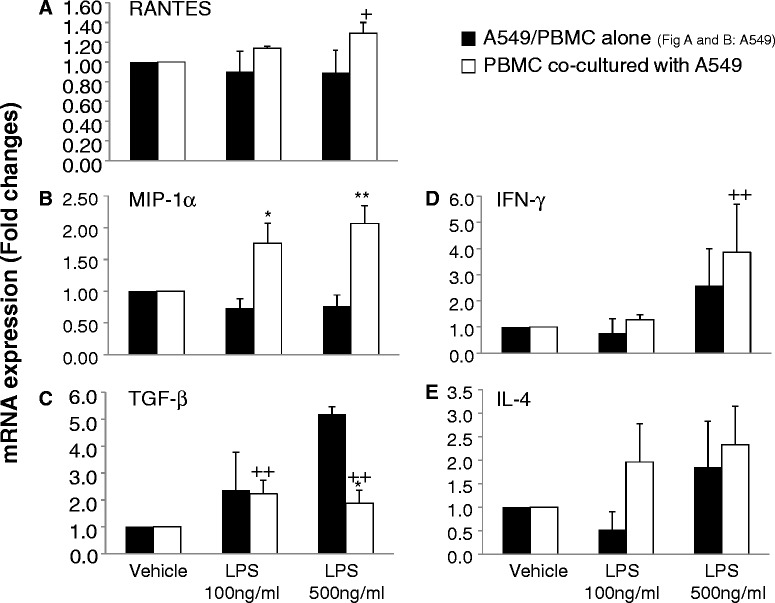
**The mRNA expressions of regulated upon activation normal T cell expressed and secreted factors (RANTES) (A) and macrophage inflammatory protein-1 alpha (MIP-1α) (B) in lung cancer cells (A549), or transforming growth factor-β (TGF-β) (C), Interferon-γ (IFN-γ) (D), and interleukin 4 (IL-4) (E) in peripheral blood mononuclear cells.** A549 cells were planted in the lower chamber of the transwell and stimulated with LPS at 100 and 500 ng/ml or vehicle for 24 hrs. PBMCs from healthy donors were then added to the upper chamber of the transwell for co-culture for 48 hrs. The control group was PBMC from healthy donors without co-culture. + and ++ stand for p values less than 0.05 and 0.01, respectively, as compared with vehicle-stimulated co-cultured A549). * and ** stand for p values less than 0.05 and 0.01, respectively, as compared with A549 at 72 h after corresponding LPS stimulation.

To investigate the role of LPS-related signal pathway in the interaction between cancer cells and immune cells, A549 cells were pretreated with or without the NF-κB inhibitor PDTC at 10, 50, 100, 300, or 500 μM for 4 hrs, followed by the stimulation of LPS at 500 ng/ml. Figure 5A demonstrated that PDTC-pretreated A549 cells significantly increased frequencies of CD4^+^T cells, while decreased frequencies of Tregs and CD45RA^+^Tregs when A549 cells were pretreated with PDTC at 300 μM (Figure 5B and C). Figure 6A and B showed a significantly increased mRNA expression of RANTES and MIP-1α in A549 in a concentration-dependent pattern after co-culture, which accompanied the up-regulation of Tregs (Figure 4C). mRNA expression of TGF-β in PBMC significantly reduced (Figure 6C), but IFN-γ and IL-4 in PBMC increased after co-culture (Figure 6D and E).

**Figure 7:**
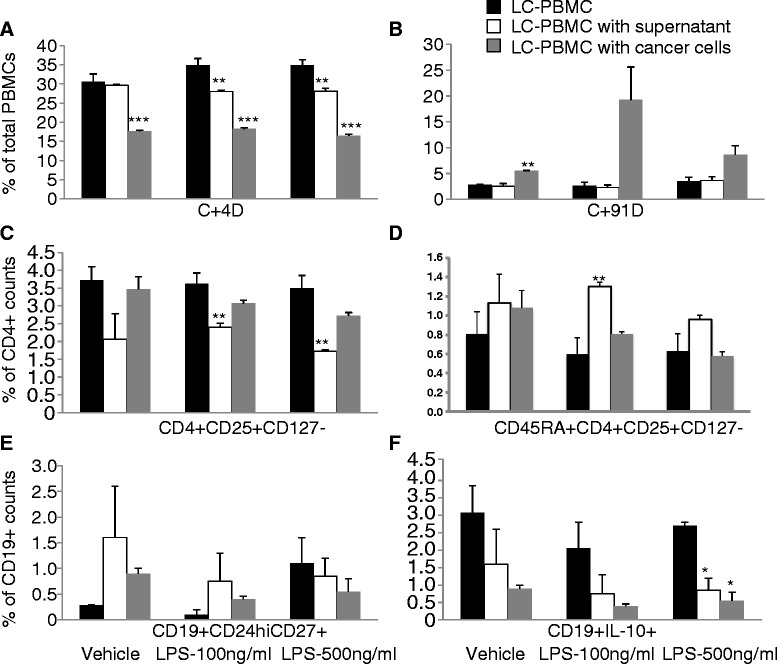
**Culture of A549 (cells and supernatant, respectively) with PBMCs from lung cancer patients.** The roles of inflammation-activated lung cancer cells in phenotype alterations of peripheral blood mononuclear cells (PBMCs) obtained from patients with lung cancer. A549 cells were stimulated with LPS at 100 and 500 ng/ml for 24 hrs, and LPS-stimulated A549 cells and their supernatant were then harvested. PBMC from lung cancer patients were co-cultured with harvested LPS-stimulated A549 cells and their supernatant for 48 hrs, respectively. The control group was PBMC from lung cancer patients without co-culture. **A**: frequency of CD4^+^ T cells in total PBMCs, **B**: frequency of CD19^+^ B cells in total PBMCs, **C**: frequency of Tregs in CD4^+^ T cells, **D**: frequency of CD45RA^+^Tregs in CD4^+^ T cells, **E**: frequency of CD19^+^CD24^hi^CD27^+^ B cells in CD19^+^ B cells, and **F**: frequency of CD19^+^IL-10^+^ B cells in CD19^+^ B cells. + stands for p value less than 0.05, as compared with PBMC co-culture with vehicle-stimulated A549 cells. *, **, and *** stand for 0.05, 0.01, and 0.001 stand for p value less than 0.05, as compared with corresponding LPS-stimulated PBMC.

Co-culture of PBMCs from lung cancer patients with A549 cells and conditioned supernatant stimulated with LPS at 100 and 500 ng/ml induced alterations in PBMC populations compared to those observed in PBMCs from healthy donors. Figure 7A demonstrated that co-culture with LPS-stimulated A549 cells or conditioned supernatant did not alter frequencies of CD4^+^T cells, but increased frequencies of CD19^+^B cells after the co-culture (Figure 7B). Frequencies of Tregs increased or decreased in conditioned supernatant stimulated with LPS at 100 or 500 ng/ml, respectively, after the co-culture. Frequencies of Tregs significantly decreased after co-culture with LPS-stimulated A549 cells in a concentration-dependent pattern (Figure 7C). Alterations in the proportion of CD45RA^+^Tregs were similar to that of Tregs, as shown in Figure 7D. Alterations of Tregs frequencies in PBMCs from lung cancer patients were mainly cell-cell-contact dependent, while alterations of CD45RA^+^Tregs were predominantly cytokine-dependent. LPS stimulation also increased the expression of cytoplasmic IL-10 in CD19^+^B cells. Frequencies of CD19^+^CD24^hi^CD27^+^ and CD19^+^IL-10^+^ B cells significantly decreased after co-culture either with LPS-stimulated A549 cells or conditioned supernatant, as compared with the control (Figure 7E and F). It seemed that alterations of Bregs were mainly cytokines dependent.

## Discussion

The immune system plays a significant role in the control of tumor progression, although the regulatory mechanism of interaction between two systems remains unclear. High proportions of Tregs were found in tumor-infiltrating lymphocytes of patients with lung cancer [7] and Tregs from patients with lung cancer directly inhibited autologous T cell proliferation [23]. The percentage of Tregs might be correlated with the pathological stage in lung cancer or tumor burden [24]. The present study demonstrated that peripheral frequencies of Tregs and CD45RA^+^Tregs in lung cancer patients was lower than those in healthy individuals, indicating a maturation-activation state of naïve Tregs and preferential homing of mature Tregs into the lungs of patients. Furthermore, the present study initially demonstrated that peripheral frequencies of Bregs cells were significantly higher in patients with lung cancer. Cancer-derived factors and the interaction of lung cancer cells with normal PBMCs may contribute to the expansion of Bregs, similar alterations of Tregs and Bregs observed in our clinical cohort.

Leukocytes within tumors play critical roles in the formation of inflammatory microenvironment and tumorigenesis, while little has been known about the potential mechanism to communicate between inflammation and cancer [25]. The present study explored the relationship between inflammation and antitumor immunity adopting an *in vitro* model based on LPS-stimulated A549 cells. Inflammation-activated lung cancer cells or their products during the pretreatment could increase the frequencies of Tregs and CD45RA^+^Tregs from normal PBMCs. It seemed that the direct interaction between cells played a more important role in alterations of Treg phenotypes than their products which were more important in CD45RA^+^Treg phenotype alterations. Furthermore, continuous LPS stimulation during the interaction between cancer cells and PMBCs could increase frequencies of Tregs and CD45RA^+^Tregs. The increase of Tregs might also result from the natural Treg self-expansion promoted by inflammatory factors or the conversion of naïve CD4^+^ T cells.

Previous study demonstrated that the normal maturation-activation process of T cells was involved in the sequential expression of naïve T cells, mature T cells, or effector/cytotoxic T cells [26]. CD45RA^+^Tregs in the periphery of humans express high levels of FOXP3 and manifest equivalent suppressive activity as compared to CD45RO^+^Tregs counterparts [27]. Our observation of a higher proportion of CD45RA^+^Tregs indicates a final maturation-activation state of those cells promoted by cancer-related inflammatory factors. Inflammation-activated cancer cells could also play the initiators and/or secondary sources of the development of cancer microenvironment and alterations of local immunity through the direct interaction and products. The present study demonstrated that NF-κB inhibition of inflammation-activated cancer cells could decrease frequencies of Tregs and CD45RA^+^Tregs. Inflammation was also found to stimulate the production of chemo-attractants from lung cancer cells, responsible for the recruitment of infiltrated inflammatory cells.

Tumor cells play a crucial role in the conversion of naïve and/or effector T cells into Treg by providing antigenic stimulation and cytokines, although little has been known on the influence of cytokines on Treg proliferation or activation during the interaction between tumor and inflammatory cells. The previous study demonstrated that overexpression of RANTES was associated with improved prognosis in lung cancer [28]. Lung cancer cells were found to produce MIP-1α which might affect the interaction between lung cancer and host inflammatory cells [29]. The present study observed that mRNA expressions of RANTES and MIP-1α in cancer cells after co-culture of cancer cells and PBMCs in a concentration-dependent pattern, accompanied with the up-regulation of Tregs.

Interaction between PBMCs and inflammation-activated cancer cells or their products also increased the frequency of CD19^+^B cells and the frequency of CD19^+^CD24^hi^CD27^+^ B cells in a LPS-concentration dependent manner. Inflammation-activated cancer cells-driven products could induce the high expression of cytoplasmic IL-10 in B cells. It seems that the influencing roles of inflammation-activated cancer cells in the frequencies of CD19^+^CD24^hi^CD27^+^ and CD19^+^IL-10^+^ B cells are associated with the severities of inflammation. The interaction between inflammation-activated cancer cells or their products with PMBCs can play a critical role in the expansion of Bregs.

On basis of our finding that co-culture led to phenotype alterations of PBMCs from healthy individuals, we further investigated the role of inflammation-activated cancer cells in PBMCs from patients with lung cancer and found similar alterations of Treg and CD45RA^+^Treg phenotypes in PBMC from lung cancer patients to those in healthy donors. However, the interaction between PBMCs from lung cancer patients with inflammation-activated cancer cells decreased the frequency of Bregs, which might be explained by the immune state of cancer patients. Growing evidence has shown interaction between Tregs and Bregs in tumor microenvironment. A previous study revealed that Bregs in the lung metastasis from breast cancer were able to induce conversion of resting CD4^+^T cells to Tregs to support metastatic growth [17]. The observation might also explain the expansion of Tregs in our co-culture-model. More investigations are needed to further explore the interactions between Tregs and Bregs and the underlying mechanism, involving mediators from both Tregs and Bregs or potential network biomarkers [30-38].

In conclusion, we found decreased or increased frequencies of peripheral Tregs or Bregs in patients with lung cancer where the direct interaction of inflammation-activated cancer cells may play the critical and dominant roles (Additional file 2: Figure S2). Effects of lung cancer cells were associated with the severity of inflammation. Further studies are needed to reveal the underlying mechanisms leading to the alterations of lymphocyte phenotypes. Strategies against regulatory lymphocytes may be potential for tumor therapy in the future.

## Additional files

Additional file 1: Figure S1. A-1D Experiment designs. Additional file 2: Figure S2. Experiment summary.
