# Supplementary material for: Enhanced frequency and potential mechanism of B regulatory cells in patients with lung cancer
Source: J Transl Med. 2014 Nov 11;12:304. doi: 10.1186/s12967-014-0304-0 (PMC4236438; doi:10.1186/s12967-014-0304-0)
Supplement: Additional file 1: Figure S1. — A-1D Experiment designs. [file 12967_2014_304_MOESM1_ESM.zip › 12967_2014_304_add1/12967_2014_304_add1.pptx]

## Slide 1
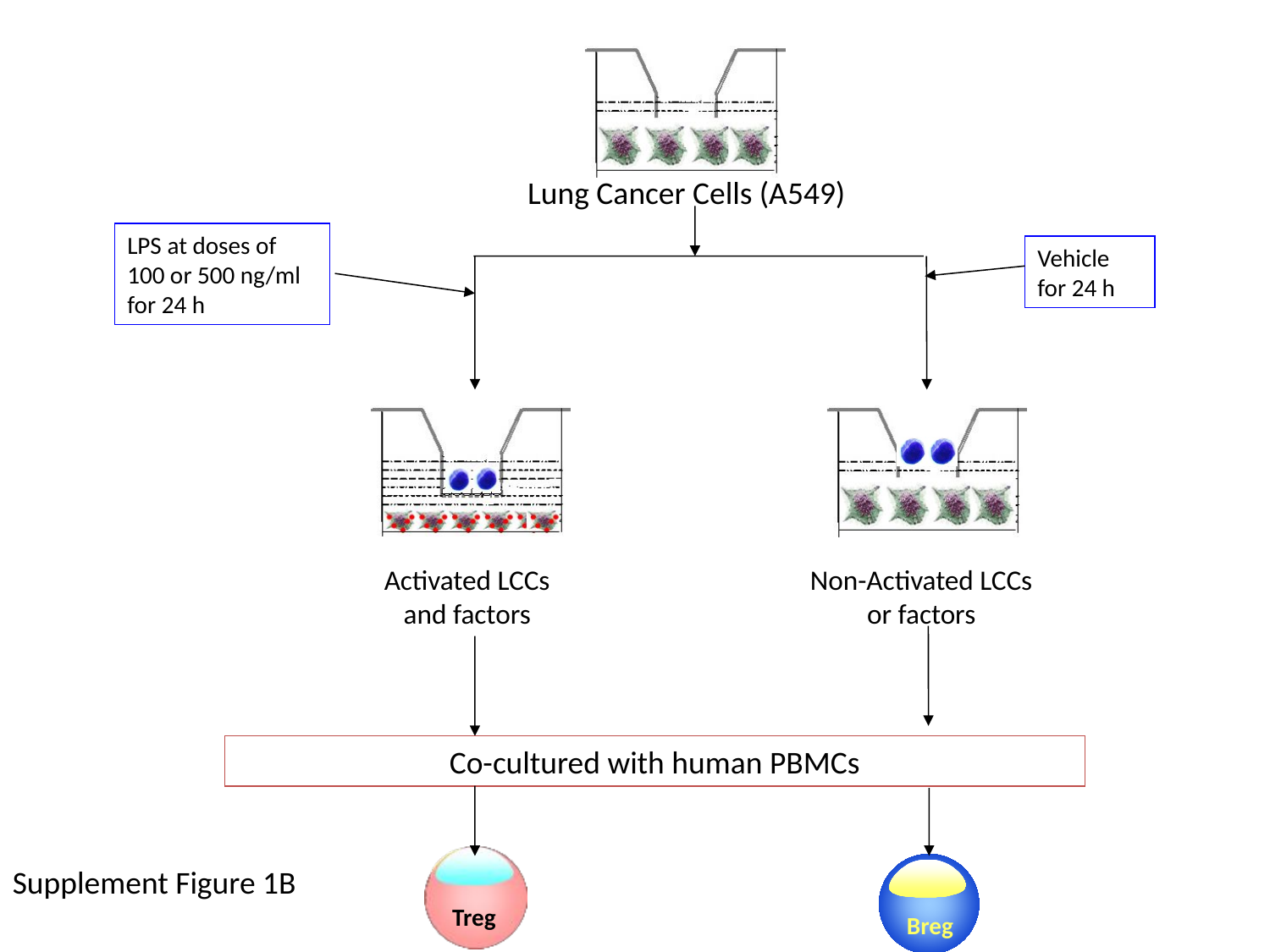

Lung Cancer Cells (A549)
LPS at doses of 100 or 500 ng/ml for 24 h
Vehicle for 24 h
Activated LCCs and factors
Non-Activated LCCs or factors
Co-cultured with human PBMCs
Breg
Supplement Figure 1B
Treg
