# Supplementary material for: Enhanced frequency and potential mechanism of B regulatory cells in patients with lung cancer
Source: J Transl Med. 2014 Nov 11;12:304. doi: 10.1186/s12967-014-0304-0 (PMC4236438; doi:10.1186/s12967-014-0304-0)
Supplement: Additional file 1: Figure S1. — A-1D Experiment designs. [file 12967_2014_304_MOESM1_ESM.zip › 12967_2014_304_add1/12967_2014_304_add2.pptx]

## Slide 1
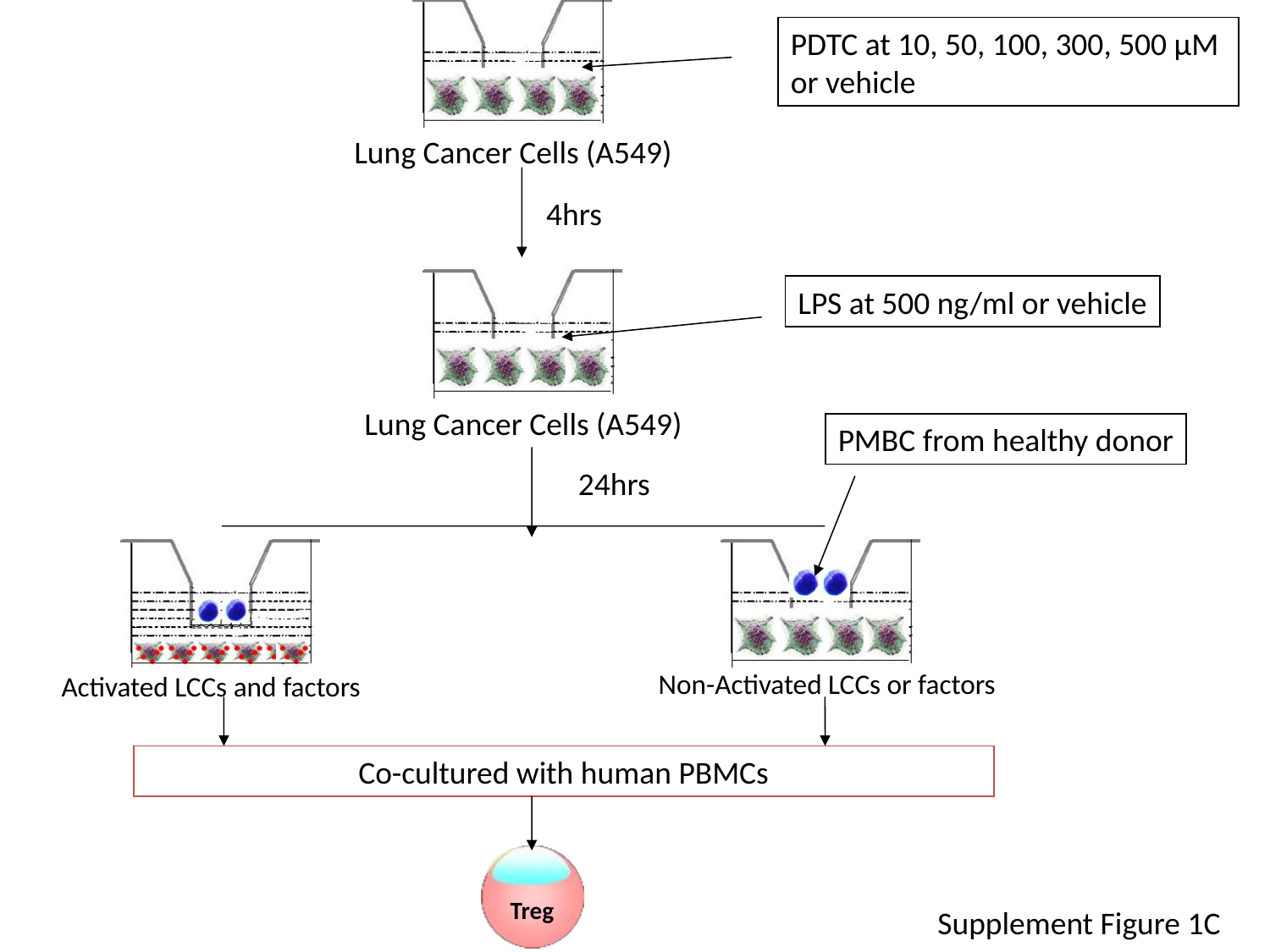

Lung Cancer Cells (A549)
PDTC at 10, 50, 100, 300, 500 μM
or vehicle
 4hrs
Lung Cancer Cells (A549)
LPS at 500 ng/ml or vehicle
PMBC from healthy donor
24hrs
Non-Activated LCCs or factors
Activated LCCs and factors
Co-cultured with human PBMCs
Treg
Supplement Figure 1C
