# Supplementary material for: Enhanced frequency and potential mechanism of B regulatory cells in patients with lung cancer
Source: J Transl Med. 2014 Nov 11;12:304. doi: 10.1186/s12967-014-0304-0 (PMC4236438; doi:10.1186/s12967-014-0304-0)
Supplement: Additional file 1: Figure S1. — A-1D Experiment designs. [file 12967_2014_304_MOESM1_ESM.zip › 12967_2014_304_add1/12967_2014_304_add4.pptx]

## Slide 1
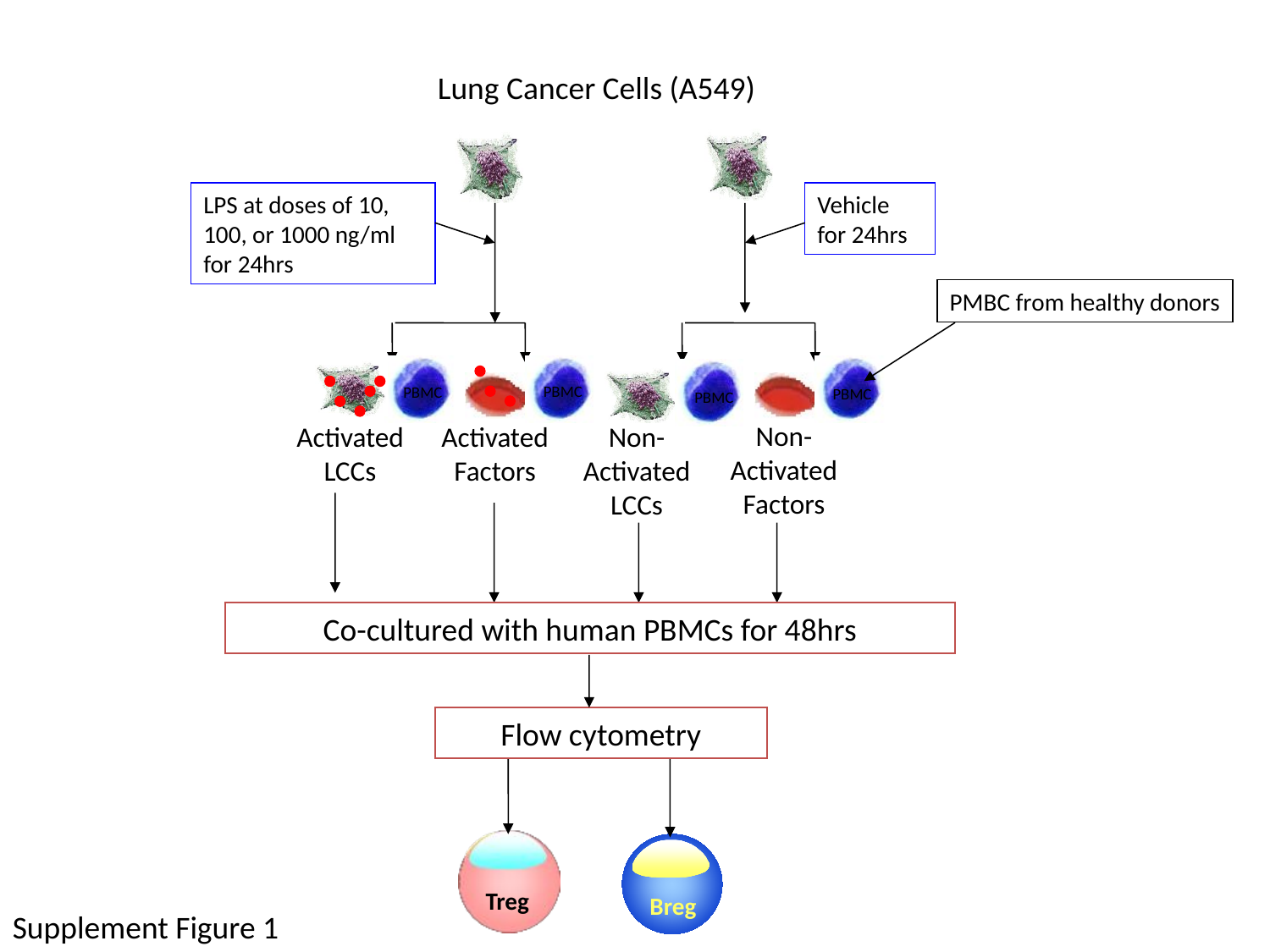

Lung Cancer Cells (A549)
LPS at doses of 10, 100, or 1000 ng/ml for 24hrs
Vehicle for 24hrs
PMBC from healthy donors
PBMC
PBMC
PBMC
PBMC
Non-Activated
Factors
Activated LCCs
Activated
Factors
Non-Activated LCCs
Co-cultured with human PBMCs for 48hrs
Flow cytometry
Treg
Breg
Supplement Figure 1
