# Supplementary figures and images for: Enhanced frequency and potential mechanism of B regulatory cells in patients with lung cancer
Source: J Transl Med. 2014 Nov 11;12:304. doi: 10.1186/s12967-014-0304-0 (PMC4236438; doi:10.1186/s12967-014-0304-0)

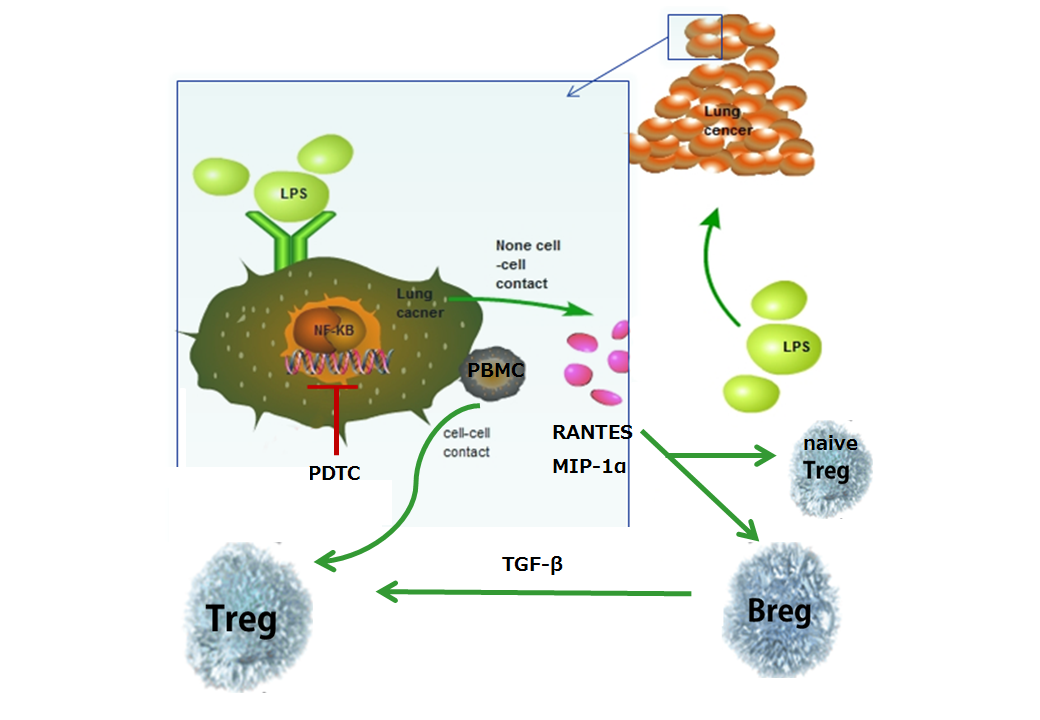

Supplement: Additional file 2: Figure S2. — Experiment summary. [file 12967_2014_304_MOESM2_ESM.tiff]
